# Prediction of enteric methane emissions from lactating cows using methane to carbon dioxide ratio in the breath

**DOI:** 10.1111/asj.13637

**Published:** 2021-09-30

**Authors:** Tomoyuki Suzuki, Yuko Kamiya, Kohei Oikawa, Itoko Nonaka, Takumi Shinkai, Fuminori Terada, Taketo Obitsu

**Affiliations:** ^1^ Institute of Livestock and Grassland Science NARO Nasushiobara Japan; ^2^ Institute of Livestock and Grassland Science NARO Tsukuba Japan; ^3^ Graduate School of Biosphere Science Hiroshima University Higashihiroshima Japan

**Keywords:** automatic milking system, dietary fiber, eating, methane conversion factor, sniffer method

## Abstract

The aim of this study was to develop prediction equations for methane (CH_4_) emissions from lactating cows using the CH_4_/carbon dioxide (CO_2_) ratio in the breath measured in the automatic milking system (AMS) and to evaluate the predicted values and factors affecting the CH_4_/CO_2_ ratio. The model development was conducted using a dataset determined in respiration chambers or head boxes (*n* = 121). Then, gas measurements in the AMS as well as in the head box were carried out with six lactating cows fed one of three different levels of neutral detergent fiber (NDF) content, following a 3 × 3 Latin square experimental design. The obtained equation that is suitable for practical use on farms to predict CH_4_ was CH_4_ (L/day) = −507 + 0.536 live weight (kg) + 8.76 energy‐corrected milk (kg/day) + 5,029 CH_4_/CO_2_ (adjusted *R* ^2^ = 0.83; root mean square error = 40.8 L/day). Results showed that the predicted values correlated positively with the observed values, the determined CH_4_/CO_2_ ratio increased with increasing dietary NDF content, and the detected eructation rate was in the normal range. On the other hand, the CH_4_/CO_2_ ratio was affected by the time interval between measurement and last eating before the measurement.

## 1 INTRODUCTION

Methane (CH_4_) has a strong impact on the global environment, with a global warming potential 28 times that of carbon dioxide (CO_2_; Myhre et al., 2013), and CH_4_ emission derived from enteric fermentation is the largest source within the agricultural sector (Gerber et al., 2013). Among the categories of farm animals, dairy cows are major players in enteric CH_4_ emissions (Steinfeld et al., 2006). Major mitigation options bases on modification of the rumen microbiome associated with CH_4_ production using inhibitors, plant bioactive compounds, dietary lipids, inclusion of concentrates, and so on (Hristov et al., 2013).

Additionally, selective breeding of cows with low CH_4_ emissions has been suggested as another promising mitigation option (Pickering et al., 2015). Genomic studies show that heritability of CH_4_ emission ranged from approximately 0.12 to 0.45 (Breider et al., 2019; Lassen & Løvendahl, 2016; Pszczola et al., 2017), with repeatability ranging from 0.25 to 0.69 (Breider et al., 2019; Negussie et al., 2017; Pszczola et al., 2017). For selective breeding of cows with low CH_4_ emissions, it is necessary to adopt a reliable and cost‐effective data collection method for the large number of cows at production sites. The method with respiration chamber or head box, which was originally used to determine heat production indirectly, provides accurate standard data; however, the method is not suitable for large amounts of data collection owing to its high construction and labor costs. The method to collect breathed air from feed bins during eating would be the most promising method for the CH_4_ data collection from large numbers of animals. This approach can be separated into two methods: In one method, all emitted CH_4_ or CH_4_ flux during eating is collected in the feed bin (ex. GreenFeed; C‐Lock Inc., Rapid City, SD, USA), and in the other method, a part of the breath during milking in the automatic milking system (AMS) or during eating in the individual feeder is collected. This latter approach is called the “sniffer method” (Garnsworthy et al., 2012). The sniffer method is separated into two methods: with or without CO_2_ measurement. One method measures only the CH_4_ concentration in the air around the feed bin and determines CH_4_ emissions using the CH_4_ concentration and its dilution factor. The other method measures CH_4_ concentration together with CO_2_ concentration as a tracer gas, in which the volume of the emitted CH_4_ is determined as the product of the CH_4_/CO_2_ ratio and daily CO_2_ emission predicted from the heat production unit (HPU) and HPU equivalent in CO_2_ (Madsen et al., 2010). The advantage of the sniffer method using CO_2_ as a tracer is that the system is simpler than flux methods such as GreenFeed, and the CH_4_/CO_2_ ratio is seldom affected by the position of the animal's head to the sample‐gas inlet.

For animal selection, correlation between predicted values and actual values is necessary, but it is not necessary to match the actual values. On the other hand, if the predicted CH_4_ emission accurately reflects the actual value, the prediction method can be utilized for the practical evaluation of CH_4_ inhibition substances at the commercial‐farm level or for checking and controlling individual daily CH_4_ emissions. Factors affecting the accuracy of the prediction using the sniffer method have been pointed out: specifically, diurnal changes in emitted CH_4_ and the validity of the prediction model. Lassen et al. (2012) showed a diurnal pattern of the CH_4_/CO_2_ ratio in lactating cows using a Fourier series approach relating to feed intake and fermentation pattern in the rumen. Brask et al. (2015) found a rapid increase and gradual decline in CH_4_ emissions from lactating cows after eating. It might be necessary to correct the obtained CH_4_/CO_2_ ratio by measurement, because the time interval between gas measurement in the AMS and feeding or eating before the measurement is not constant.

The prediction method using the HPU, proposed by Madsen et al. (2010), does not consider the variation in energy‐utilization efficiency and body‐fat mobilization, which would cause variations in CH_4_ emissions according to the individual feed efficiency or lactation period (Huhtanen et al., 2015). Additionally, Hellwing et al. (2013) found an underestimation of the predicted heat production and consequently of CH_4_ emissions, as well, using the HPU method for a dataset of lactating cows, collected in the respiration chamber. Apart from the prediction using the CH_4_/CO_2_ ratio, there are many types of prediction equations for CH_4_ emissions by animal species, breed, production stage, or region, mainly for the purpose of constructing a greenhouse‐gas‐emission inventory (Moraes et al., 2014; Ramin & Huhtanen, 2013; Shibata et al., 1993; Yan et al., 2009). These equations were constructed with the amount of nutrient intake and/or nutrient concentrations of the feed as explanatory variables. If the range of CH_4_ emissions and values for explanatory variables in the dataset for model development were sufficiently wide, the multiple regression equation would also be adaptable for the direct prediction using the CH_4_/CO_2_ ratio without fixed HPU equivalent in CO_2_ and without considering energy utilization efficiency or body‐fat mobilization.

To evaluate the prediction methods for CH_4_ emissions from lactating cows using the sniffer method in the AMS with CH_4_/CO_2_ ratio, firstly, the prediction equations were built using datasets from the trials that determined gas emissions in the respiration chambers or head boxes that apply a similar principle for measurement as respiration chambers do. Secondly, measurements and predictions of CH_4_ emissions were conducted using the head box together with the sniffer method in the AMS by lactating cows fed partially mixed ration (PMR) with different levels of dietary neutral detergent fiber (NDF), a major dietary factor affecting CH_4_ emissions. This measurement in the head box was carried out under the similar condition as the AMS, to compare estimated CH_4_ emission with actual emission in the AMS. Using data from the second trial, the CH_4_ emissions derived from the built equations and the effect of the time interval between eating and gas measurements were evaluated.

## 2 MATERIALS AND METHODS

### 2.1 Metadata analysis

Individual data (*n* = 121) of Holstein lactating cows from nine experiments covering 18 different diets were used for model development. All data were collected with an open‐circuit indirect calorimetry apparatus with a whole‐body chamber or a head box at the NARO Institute of Livestock and Grassland Science (Iwasaki et al., 1982) or whole‐body chamber at the NARO Kyushu Okinawa Agricultural Research Center (Kurihara et al., 1989; Suzuki et al., 2012). Each gas measurement was carried out continuously for 3 or 5 days, following 9–18 days of adaptation. Average daily CH_4_ emissions of individual cows during the measurement period were used for the analysis. Temperature and humidity in the chambers or in the experimental room equipped with head boxes were fixed at 18°C and 60%, 28°C and 40%, or 28°C and 60%, or controlled at 24–32°C and 40%–80% during the day. The cows were fed a typical roughage and concentrate available in Japan. A general description of the dataset is provided in Table S1.

Single and multiple regression equations were developed using the generalized linear model of JMP 15.2.1 software (SAS Institute Inc., Cary, NC, USA). The dependent variables were CH_4_ emission (L/day) and CH_4_ conversion factor (MCF, joules [J]/100 J gross energy intake [GEI]). The independent variables were dry matter intake (DMI kg/day), live weight (LW, kg), energy‐corrected milk yield (ECM), and CH_4_/CO_2_ ratio (L/L). The ECM was calculated in accordance with the equation by Tyrrell and Reid (1965); ECM (kg/day) = milk yield (kg/day) × (376 × milk fat [%] + 209 × milk protein [%] + 948)/3,138. The CH_4_/CO_2_ ratio was calculated using the daily CH_4_ and CO_2_ emissions (L/day). To develop a useful prediction equation for commercial farms, the effects of temperature, humidity, experiment, and animal were treated as residual errors. Correlations among the independent variables were determined using the JMP 15.2.1 software.

Additionally, according to the method by Madsen et al. (2010), CH_4_ emissions were predicted using the CH_4_/CO_2_ ratio and the CO_2_ emissions derived from the multiplication of HPU equivalent in CO_2_ (180 L/h/HPU [×10^3^ W]; Pedersen et al., 2008) and HPU. The HPU was calculated using the ECM, LW, and days in pregnancy: HPU (×10^3^ W) = (5.6 × LW^0.75^ [kg] + 22 × ECM [kg/day] + 1.6 × 10^−3^ × days in pregnancy)/10^3^ (Internationale Commision du Génie Rural [CIGR], 2002). Finally, daily CH_4_ emissions (L/day) were calculated as CH_4_ = CH_4_/CO_2_ ratio × 180 × HPU × 24. Due to the lack of data on the days in pregnancy for each cow, pregnancy length was estimated using an open period of 126 days, which was defined as subtracting 280 days of gestation length from 406 days of a median calving interval in Japan (Livestock Improvement Association of Japan Inc., 2021). The days in pregnancy of cows before 126 days in milk was defined as 0 days, while that of cows after 126 days in milk was defined as subtracting 126 days from the days in milk. The association between the observed and estimated CO_2_ and CH_4_ emissions derived from the equation including HPU was examined using the linear regression procedure of the JMP 15.2.1 software.

### 2.2 Evaluation of sniffer method and developed models

#### 2.2.1 Feeding trial

All animal studies were conducted in accordance with the animal care and use guidelines of the NARO (Approved no. 1811B040).

This experiment was carried out in a free‐stall barn equipped with an individual door feeder and two boxes of AMS with one‐way traffic (MIone, GEA Farm Technologies GmbH, Siemensstraße, Germany) in the experimental barn of the Institute of Livestock and Grassland Science, NARO. Six multiparous Holstein cows (3.0 ± 1.2 [mean ± SD] parity, 647 ± 36.7 kg of initial body weight, 97 ± 30.8 days in milk) were randomly assigned to a 3 × 3 Latin square design with three dietary treatments of different NDF level (low‐fiber [LF], medium‐fiber [MF], or high‐fiber [HF]). Each experimental period was 21 days, consisting of a 16‐day adaptation period and a 5‐day data‐collection period. The PMRs were formulated to provide three levels of NDF content (Table 1). The NDF content was determined by adjusting the forage‐to‐concentrate ratio and the proportion of forage, including timothy hay, whole‐crop rice silage, and corn silage. Commercial concentrate and soybean meal were used at the same levels of crude protein (CP) and ether extract.

**TABLE 1:** Formula and chemical composition of partially mixed rations (PMRs) for low‐fiber (LF), medium‐fiber (MF), and high‐fiber (HF) dietary treatments, as well as commercial concentrate provided in the automatic milking system

	PMR			Commercial concentrate
	LF	MF	HF	
Formula (DM basis)				
Timothy hay, %	0.0	23.2	47.1	—
Whole crop rice silage, %	25.1	15.2	5.1	—
Corn silage, %	17.4	8.8	0.0	—
Unhulled rice silage, %	19.5	19.7	20.0	—
Commercial concentrate, %	32.8	24.2	15.4	—
Soybean meal, %	4.9	8.4	12.1	—
Vitamin and mineral mix, %	0.4	0.4	0.4	—
Chemical composition				
DM, %	45.5	58.0	72.2	87.1
Organic matter, %DM	92.1	92.5	93.1	94.0
Ether extracts, %DM	2.32	2.08	1.72	3.35
Crude protein, %DM	13.7	14.2	12.5	19.8
NDF, %DM	31.6	37.0	47.1	15.9
ADF, %DM	19.1	22.5	28.3	7.7
NSC, %DM	44.4	39.3	31.8	55.0
Starch, %DM	33.0	26.4	17.5	40.1
Gross energy, kJ/gDM	18.4	18.5	18.6	18.7

Abbreviations: ADF, acid detergent fiber; DM, dry matter; kJ, kilojoule; NDF, neutral detergent fiber; NSC, non‐structural carbohydrates.

Daily milking at the AMS was permitted between 05:00 and 09:00 h and between 17:00 and 18:00 h, so that all cows were milked twice daily. Cows were offered one of three PMRs at each door feeder (allowing approximately 10% refusals) three times daily (10:00, 13:00, and 17:00 h). Additionally, the cows were offered 0.33 kg (as feed) of commercial concentrate (Nyuhai‐YAWARA; JA Higashi‐Nihon Kumiai Shiryo, Otawara, Japan) at the 13:00‐h feeding and at each milking in the AMS. Therefore, the cows consumed a total of 1.0 kg (as feed) of commercial concentrate daily. Water and mineral blocks (Koen; Nippon Zenyaku Kogyo Co. Ltd., Koriyama, Japan) were freely accessed.

During the last 5 days of each experimental period, the amount of refusals was weighed, and representative samples were collected daily before feeding at 10:00 h and kept in the refrigerator. Each daily sample of refusals was mixed for cows in each period, and the samples were collected. The samples of offered diets and refusals were dried at 60°C in an air‐forced oven for 48 h, and then ground through a 1‐mm mesh using a Wiley mill (1029‐C; YOSHIDA SEISAKUSHO CO., LTD., Tokyo, Japan) for further analysis. Stored data for individual milk yields were collected from the AMS. Milk samples were automatically collected from the evening of Day 17 to the morning of day 21. The LW of cows was measured before morning feeding on the initial day of the experiment, then on Days 7 and 21 in each experimental period. Rumen fluid was collected orally using a catheter (FUJIHIRA INDUSTRY Co., Ltd., Tokyo, Japan) before the morning feeding on the last day of each experimental period and filtered through 4 layers of cheese cloth; pH was determined using a glass‐electrode pH meter (D‐210P; HORIBA Advanced Techno, Co., Ltd., Kyoto, Japan), after which the fluid was stored at −40°C for lab analysis.

The time spent eating and ruminating by each cow was recorded using a neck‐tag device, including an accelerometer and a barometric pressure sensor (U‐motion; Desamis Co., Ltd., Tokyo, Japan). The device detects eating, ruminating, moving, standing, or lying, and then adds up time spent on those activities during each 10 min; the activity with the most time during the 10 min was defined as the dominant activity of each 10 min. The chewing time of each cow was recorded every 10 min and used for analysis.

#### 2.2.2 Measurements in the AMS

Gas collection during milking in the AMS was carried out on Days 17–21 of each experimental period. Air around the feed bin in the AMS was vacuumed with the pump (approximately 6.5 L/min). Filtrated and dehumidified sample gas was sent to the infrared CH_4_ and CO_2_ analyzers (ZRF; Fuji Electric Co., Ltd., Tokyo, Japan) or a multigas infrared analyzer (PG‐300, HORIBA, Ltd., Tokyo, Japan). Layout of the gas collection system is provided in Diagram S1. The signals from the gas analyzers were converted to gas concentrations every 1 s with an I/O controller (CPU‐CA20(FIT)GY; Contec CO., Ltd., Osaka, Japan). The analyzers were calibrated daily using nitrogen (99.999%), a nitrogen‐balanced CH_4_ (0.193%) mixture, and a nitrogen‐balanced CO_2_ (1.92%) mixture. To obtain the background gas concentration, measurements were performed for 5 min before or after milking. The average CH_4_ and CO_2_ concentrations during a 5‐min period were used for background correction. Additionally, to eliminate data during which the cow's head was far from the feed bin, CH_4_ and CO_2_ data in each 1 s were removed if the difference between the detected CO_2_ concentration and background concentration was lower than 500 ppm. After background correction (sample gas concentration minus background gas concentration), CH_4_ and CO_2_ were averaged at each visit, and consequently, the CH_4_/CO_2_ ratio was obtained for the CH_4_ emission prediction. Separately, the eructation rate, defined as the peak number of CH_4_/CO_2_ ratio per minute calculated with CH_4_/CO_2_ ratio every 1 s during the visit, was determined with a custom Python script using the SciPy module (http://www.scipy.org/).

#### 2.2.3 Measurements in the head box

The CH_4_ and CO_2_ emissions from cows were measured in two individual tie‐stalls (L1600 × W1170 mm) equipped with a ventilated caloriemeter using a head box (W980 × D900 × H2030 mm) in the experimental barn located next to the free‐stall barn. During 13:30 and 15:00 h of Days 18 and 21 of each experimental period, the cows were brought to the tie‐stall, and their gas emissions were measured for 15 min. To be a similar condition to the gas measurement in the AMS, cows were fed 0.33 g (as fed) of concentrate during measurement in the head box, whereas not fed the concentrate at the 13:00‐h feeding. The measurements were repeated four times during each period for each cow. The cows were returned to the free‐stall barn immediately after the measurement.

The gas in the head box was vacuumed with a blower at approximately 520 L/min. The gas‐flow rate was determined using a thermal flowmeter (NFHY‐R; Nippon Flowcell Co. Ltd., Tokyo, Japan). Sample gas was collected from vacuumed gas and sent to the CH_4_ and CO_2_ analyzers (ZRF; Fuji Electric Co., Ltd.) via a filter and dryer. The signals from the flowmeter and gas analyzers were converted to the flow rate and gas concentration with the I/O system (CPU‐CA20(FIT)GY; Contec CO., Ltd.) every 1 s. CH_4_ and CO_2_ emissions per minute were calculated by multiplying the vacuum gas flow (L/min, at 0°C, 1 atm) by the difference between the gas concentrations in the vacuumed air and those in the background gas that was measured before the gas measurement of cows. The total volume of emitted CH_4_ and CO_2_ during 15 min was used to calculate the CH_4_/CO_2_ ratio. Daily CH_4_ emissions were defined as the product of 1440/15 and total CH_4_ emissions over 15 min. The analyzers were calibrated daily using nitrogen (99.999%), a nitrogen‐balanced CH_4_ (0.193%) mixture, and a nitrogen‐balanced CO_2_ (1.92%) mixture. Prior to the experiment, the CO_2_ recovery test was conducted three times using 99.999% CO_2_ gas. The recovery rates of the two head boxes were 102.0% ± 1.57% and 100.5 ± 1.76%. The principles of the head box system, measurement, and calibration have been described elsewhere (Suzuki et al., 2007).

#### 2.2.4 Chemical analysis

The dry matter (DM, 135°C for 2 h) and chemical components of the diets and refusals were determined. The ether extract, Kjeldahl N, and crude ash values were determined according to Association of Official Analytical Chemists (AOAC) (2000; methods 920.39, 990.03, and 942.05, respectively). The organic matter (OM) was calculated as weight loss during ashing. The NDF was assayed with a heat‐stable amylase and sodium sulfite, and expressed exclusive of residual ash. This and acid detergent fiber (ADF) expressed exclusive of residual ash were analyzed according to the methods of van Soest et al. (1991) and the AOAC (2000; method 973.18). Starch was assayed using a commercial kit (Total Starch Assay Kit; Megazyme Ltd., Wicklow, Ireland). The gross energy was determined using an adiabatic bomb calorimeter (CA‐4PJ; Shimadzu, Kyoto, Japan). Short‐chain fatty acids (SCFAs) in the rumen fluid were determined using a gas chromatograph (6890; Hewlett‐Packard, Palo Alto, CA, USA) with a glass column packed with 5% Thermon 1000 and 0.5% H_3_PO_4_ on 80/100 mesh Chromosorb W (Wako Pure Chemical Ltd., Osaka, Japan). Milk samples were analyzed for fat, protein, and lactose concentrations by infrared spectroscopy (Milko‐Scan 133B; N. Foss Electric, Hillerød, Denmark).

#### 2.2.5 Statistical analysis

All data and CH_4_ emission estimates from prediction equations using variables obtained from the present feeding trial were analyzed using the MIXED procedure of SAS. The model included the fixed effect of diet treatment and the random effects of square and cow within the square. The linear mixed model was used for the analysis of CH_4_/CO_2_ ratio, CH_4_ and CO_2_ concentrations, and eructation rate, which were collected in the AMS, using the MIXED procedure with the fixed effect of time interval between the measurement in the AMS and the end of the last eating before measurement, interaction of the time interval and diet treatment, diet treatment, and the random effect of square and cow within square. For those analyses, the covariance structure that resulted in the lowest Akaike's information criterion was chosen. The main effects of diet were examined using Tukey's multiple range test. Significance was declared at *p* < 0.05 and tendency when 0.05 ≤ *p* < 0.10. The association between observed and predicted CH_4_ emissions, MCF, and CH_4_/CO_2_ ratio was examined using the REG procedure in the SAS software.

## 3 RESULTS AND DISCUSSION

### 3.1 Model development for CH_4_ emissions

The mean ± SD of days in milk, DMI, ECM, CH_4_/CO_2_, and CH_4_ emission in dataset were 147 ± 69.1 day, 16.7 ± 3.68 kg/day, 27.4 ± 6.78 kg/day, 0.088 ± 0.0119, and 471 ± 99.9 L/day, respectively (detailed in Table S2). The MCF of the dataset ranged from 3.40 to 7.98 J/100 J GEI. The average MCF of 6.12 J/100 J GEI was close to the IPCC (2006) default value of 6.5 J/100 J GEI for non‐fattening cattle.

Significant (*p* < 0.05) correlations between independent variables for model development were found between LW and DMI (*r* = 0.51), LW and ECM (*r* = 0.32), and DMI and ECM (*r* = 0.80). The fitness of the model consisting of the DMI as the sole independent variable (Equation 7 in Table 2) was much higher than that of the model consisting of the ECM (Equation 6). When the LW or CH_4_/CO_2_ ratio was selected as the second independent variable, model improvement from adding the CH_4_/CO_2_ ratio (Equations 4 and 5) was higher than that from adding LW. The models including LW and DMI (adjusted *R* ^2^ = 0.652, RMSE = 58.9 L/day) and including LW and ECM (adjusted *R* ^2^ = 0.471, RMSE = 72.7 L/day) as variables were not shown, because of a little model improvement, considering practical utility. Moreover, the addition of LW as an independent variable to Equations 4 and 5 resulted in an improvement in *R* ^2^ and a decrease in the RMSE (Equations 2 and 3). The model with the highest fitness was the model with ECM added to Equation 3 (Equation 1), but the improvement in the adjusted *R* ^2^ was small because of the strong correlation between the DMI and ECM. The highest variance inflation factor (VIF) of the variables in Equation 1 was 3.8 for DMI, which was slightly higher than the VIFs in all other equations for CH_4_ emissions (<1.4). The volume of CH_4_ emission is primarily affected by the DMI (Hristov et al., 2013), which is positively correlated with ECM or LW. Therefore, it is reasonable that the coefficients of DMI, ECM, and LW in the prediction equations were positive.

**TABLE 2:** Regression equations for predicting daily CH_4_ emission and for CH_4_ conversion factor (MCF) in Holstein lactating cows

Equation		Adjusted *R* ^2^	RMSE
CH_4_ (L/day)			
$\;=-397\;\left(37.4\right)+0.317\;\left(0.0557\right)\;\mathrm{LW}\;\;+13.3\;\left(1.53\right)\;\mathrm{DMI}+3.14\;\left(0.816\right)\;\mathrm{ECM}\;\;+4,343\;\left(258.1\right)\;{\mathrm{CH}}_{4}/{\mathrm{CO}}_{2}$	(1)	0.898	31.9
$=-507\;\left(45.0\right)+0.536\;\left(0.0635\right)\;\mathrm{LW}\;\;+8.76\;\left(0.630\right)\;\mathrm{ECM}+5,029\;\left(313.8\right)\;{\mathrm{CH}}_{4}/{\mathrm{CO}}_{2}$	(2)	0.833	40.8
$=-346\;\left(37.0\right)+0.277\;\left(0.0579\right)\;\mathrm{LW}\;\;+18.0\;\left(0.98\right)\;\mathrm{DMI}+4,040\;\left(259.8\right)\;{\mathrm{CH}}_{4}/{\mathrm{CO}}_{2}$	(3)	0.886	33.8
$\;=-248\;\left(41.4\right)+10.5\;\left(0.75\right)\;\mathrm{ECM}\;\;+5,169\;\left(395.7\right)\;{\mathrm{CH}}_{4}/{\mathrm{CO}}_{2}$	(4)	0.734	51.5
$\;=-219\;\left(27.9\right)+20.4\;\left(0.92\right)\;\mathrm{DMI}\;\;+3,991\;\left(282.7\right)\;{\mathrm{CH}}_{4}/{\mathrm{CO}}_{2}$	(5)	0.864	36.8
$\;=230\;\left(30.5\right)+9.54\;\left(1.168\right)\;\mathrm{ECM}$	(6)	0.354	80.2
$\;=109\;\left(25.4\right)+21.7\;\left(1.49\right)\;\mathrm{DMI}$	(7)	0.638	60.1
MCF (J/100 J)			
$=0.01\;\left(0.005\right)+0.00004\;\left(0.000008\right)\;\mathrm{LW}\;\;-0.002\;\left(0.0002\right)\;\mathrm{DMI}+0.0003\;\left(0.00011\right)\;\mathrm{ECM}\;\;+60.7\;\left(3.55\right)\;{\mathrm{CH}}_{4}/{\mathrm{CO}}_{2}$	(8)	0.766	0.440
$=1.44\;\left(0.493\right)+0.00352\;\left(0.000772\right)\;\mathrm{LW}\;\;-0.148\;\left(0.0131\right)\;\mathrm{DMI}+57.9\;\left(3.46\right)\;{\mathrm{CH}}_{4}/{\mathrm{CO}}_{2}$	(9)	0.755	0.450
$\;=2.91\;\left(0.460\right)-0.0498\;\left(0.00836\right)\;\mathrm{ECM}\;\;+51.0\;\left(4.39\right)\;{\mathrm{CH}}_{4}/{\mathrm{CO}}_{2}$	(10)	0.604	0.572
$\;=3.06\;\left(0.370\right)-0.118\;\left(0.0121\right)\;\mathrm{DMI}\;\;+57.3\;\left(3.74\right)\;{\mathrm{CH}}_{4}/{\mathrm{CO}}_{2}$	(11)	0.713	0.487
$\;=1.43\;\left(0.440\right)+53.5\;\left(4.96\right)\;{\mathrm{CH}}_{4}/{\mathrm{CO}}_{2}$	(12)	0.490	0.649

*Note*: *n* = 121. Values in parentheses indicate standard error.

Abbreviations: CH_4_/CO_2_, CH_4_/CO_2_ ratio (L/L); DMI, dry matter intake (kg/day); ECM, energy‐corrected milk (kg/day); LW, live weight (kg); MCF, CH_4_ energy in joules (J) per 100 J of gross energy intake; RMSE, root mean square error.

**FIGURE 1:**
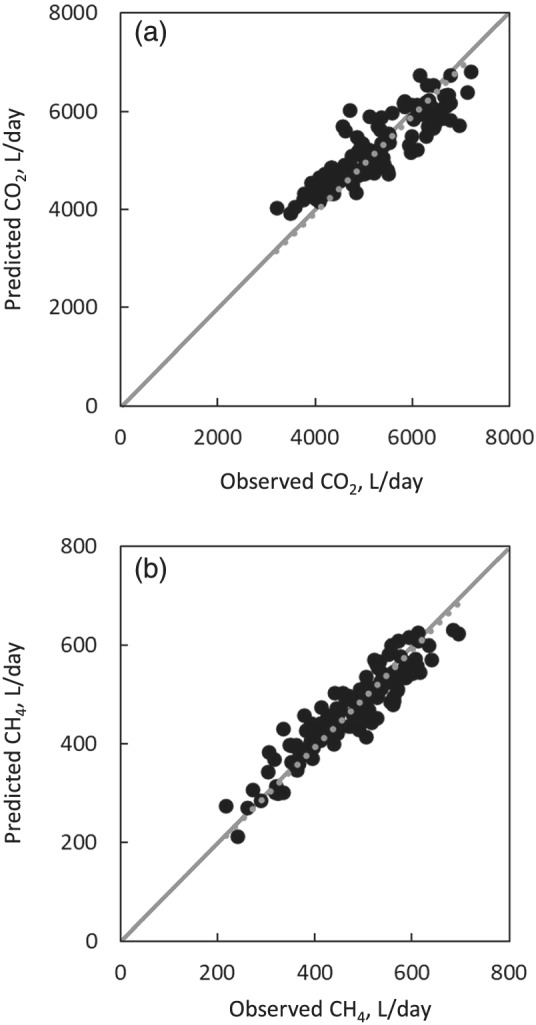
Relationships between observed CO_2_ and predicted values (a) derived from heat production unit (HPU; Internationale Commision du Génie Rural [CIGR], 2002) and HPU equivalent in CO_2_ (Pedersen et al., 2008), and between observed CH_4_ and predicted values using the equation with predicted CO_2_ and CH_4_/CO_2_ ratio (Madsen et al., 2010) (b). The equations were Y = 0.98X (adjusted *R* ^2^ = 0.99, *p* < 0.01, RMSE = 461, *n* = 121) for CO_2_ and Y = 0.98X (adjusted *R* ^2^ = 0.99, *p* < 0.01, RMSE = 39.2, *n* = 121) for CH_4_

**FIGURE 2:**
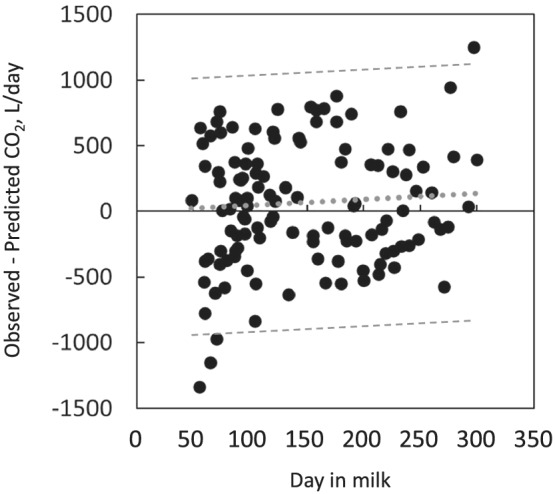
Plot of residuals (observed ‐ predicted) versus days in milk. The grey dotted line shows the regression equation; Y = 0.97X–94.8 (adjusted *R* ^2^ = 0.012, *p* = 0.17, RMSE = 467, *n* = 121). The grey dashed line shows 95% prediction interval

As a result of the comparison between observed and predicted CO_2_ or CH_4_ according to the method of Madsen et al. (2010), a significant regression with high *R* ^2^ was found and its slope was very close to 1 (Figure 1). There is a possibility of overestimation of CO_2_ emissions from cows in early lactation, in which body‐fat mobilization occurs frequently, because the HPU equivalent in CO_2_ is fixed at 180 L/h/HPU (×10^3^ W) in the prediction equation, although a lower value of 174 L/h/HPU was reported for cows in the early lactation period (Pedersen et al., 2008). A slight elevation of residuals (observed–predicted) of CO_2_ emission prediction with the progress of days in milk was found, and residuals out of the 95% prediction interval were found in the early days in milk (Figure 2), suggesting the possibility of overestimation of CO_2_ emissions due to using fixed higher HPU equivalent in CO_2_ in the early lactation period.

Equation 2 or the equation using HPU was considered most suitable for CH_4_ emission prediction in the equations, because both show higher model fitness than do other equations, and neither includes DMI as a variable, the determination of which on farms requires much more effort compared with other variables. Additionally, Equation 2 has the advantage that there is no need to determine the days in pregnancy, which is not available before pregnancy diagnosis, compared with the equation using HPU.

### 3.2 Evaluation of sniffer method and developed models for CH_4_ emissions

The dietary NDF contents of LF, MF, and HF diets were 31.6%, 37.0%, and 47.1% (DM basis), respectively, for PMR, and 31.2%, 36.5%, and 46.3%, respectively, for PMR plus concentrate in the AMS (Table 1). The NDF intake increased with increasing NDF content in the PMR, whereas dietary treatment did not influence the DMI or GEI (Table 3). The higher molar proportion of acetate and lower proportion of propionate for HF compared with LF resulted in a higher acetate‐to‐propionate ratio for HF. Dietary treatment had no effect on milk yield or ECM yield, whereas the milk fat content of cows fed the HF diet was higher than that of cows fed the LF diet. The observed CH_4_ emissions determined with the head box from cows fed LF were lower or tended to be lower than those of MF (*p* = 0.049) and HF (*p* = 0.053). Similar to the results of CH_4_ emission, the observed MCF was lower for cows fed the LF diet than for cows fed the HF diet. Daily CH_4_ emissions in the present study that were obtained from extension of measurement during only 15 min immediately after eating would be higher than actual daily CH_4_ emissions, which were measured over 24 h. In fact, CH_4_ emissions in the head box were higher than the predicted CH_4_ emissions using equations including GEI, dietary NDF and EE content, LW, and milk fat content as individual variables by Moraes et al. (2014); the predicted CH_4_ values for LF, MF, and HF were 596, 640, and 650 L/day, respectively.

**TABLE 3:** Live weight, intake, duration of chewing, ruminal parameters, milk yield and milk composition, and CH_4_ emissions of Holstein cows fed low‐fiber (LF), medium‐fiber (MF), or high‐fiber (HF) diets

	LF	MF	HF	SEM	*p* value
Number of cows	6	6	6		
Live weight, kg	692	684	694	23.4	0.195
DM intake, kg/day	25.8	26.9	25.5	1.37	0.541
NDF intake, kg/day	8.1^c^	9.9^b^	12.0^a^	0.53	<0.001
GE intake, MJ/day	474	499	473	26.0	0.497
Duration of chewing, min/kg DM intake					
Eating	13.8^b^	13.4^ab^	15.4^a^	2.34	0.037
Eating + rumination	31.9	31.0	32.2	3.37	0.746
Ruminal parameters					
Acetate, Mol%	49.3^b^	63.6^a^	65.5^a^	6.34	0.011
Propionate, Mol%	20.3^a^	19.1^a^	15.9^b^	3.76	0.011
Acetate to propionate ratio	2.7^b^	3.6^a^	4.2^a^	0.41	0.001
Ammonia N, mg/100 ml	10.0	10.6	8.8	1.13	0.074
Milk yield, kg/day	38.3	38.9	36.0	3.07	0.230
ECM yield, kg/day	35.6	36.3	34.3	2.90	0.530
Milk composition					
Fat, %	3.41^b^	3.51^ab^	3.74^a^	0.225	0.010
Protein, %	3.25	3.23	3.17	0.155	0.479
Lactose, %	4.49	4.47	4.37	0.422	0.422
Observed CH_4_ †, L/day	618^b^	768^a^	765^ab^	50.1	0.033
Observed MCF, J/100 J GEI	5.15^b^	6.17^a^	6.42^a^	0.306	0.008

*Note*: Values with different superscripts differ significantly (*p* < 0.05).

Abbreviations: AMS, automatic milking system; DM, dry matter; ECM, energy‐corrected milk; MCF, CH_4_ conversion factor (CH_4_ energy in joules [J] per 100 J of gross energy intake GEI]); MJ, megajoules; N, nitrogen; NDF, neutral detergent fiber; SEM, standard error of the mean.

^†^ Observed in head box.

**FIGURE 3:**
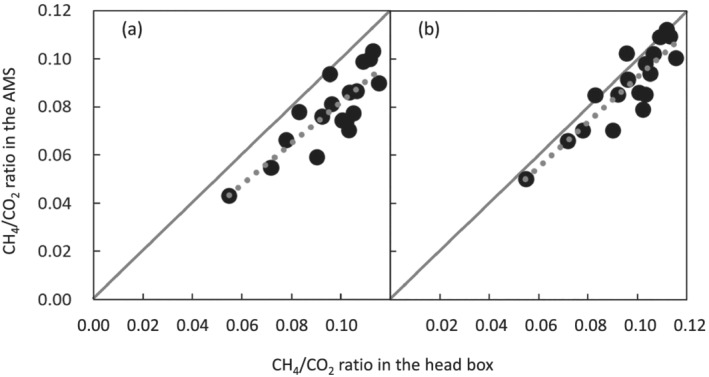
Relationships between CH_4_/CO_2_ ratio determined in the head box (X) and in the AMS (Y; *n* = 18). CH_4_/CO_2_ ratio in the AMS was used in (a). CH_4_/CO_2_ ratio in the AMS of which time interval after eating was adjusted to 0 h, was used in (b). The equations were Y = 0.81X (a) (adjusted *R* ^2^ = 0.93, *p* < 0.01, RMSE = 0.021) and Y = 0.92X (b) (adjusted *R* ^2^ = 0.93, *p* < 0.01, RMSE = 0.019)

For all diet treatments, eating was activated immediately after feeding at 10:00, 13:00, and 17:00 h, resulting in more active eating during the daytime than at night. Additionally, there were two milder peaks of eating activity at approximately 05:00 h, which were activated by milking, and at 22:00 h (Figure S1). Permission of milking in the morning was during 05:00–09:00 h, but 83% of cows were milked by 07:00 h, and all the cows were finished by 8:00 h. The peaks of average eating time at around 17:00 and 05:00 h show the eating of the cows after milking. To evaluate the effects of diet treatment and time interval between the end of last eating and gas measurement in the AMS on CH_4_/CO_2_ ratio, background‐corrected CH_4_ and CO_2_ concentrations, and eructation rate, linear mixed‐effects models were computed (Table 4). Diet treatment affected CH_4_ concentration, whereas CO_2_ concentration was not affected, resulting in a higher CH_4_/CO_2_ ratio of cows fed the HF diet compared with that of cows fed MF or LF. The model showed that the CH_4_ concentration decreased with increasing time interval between the measurement (milking) and the end of last eating before the milking, whereas CO_2_ concentration did not change with time after eating, resulting in a decrease in the CH_4_/CO_2_ ratio with time after eating. These decreasing ratios were not affected by the diet treatment. This effect of the time interval after eating would result in a lower CH_4_/CO_2_ ratio in the AMS compared with that determined in the head box (Figure 3a); the CH_4_/CO_2_ ratio in the AMS was 81% of that in the head box, from the slope of the linear regression equation passing through the origin. The mean time interval between the end of last eating before measurement in the AMS for cows fed LF, MF, and HF diets were 2.8, 3.6, and 5.2 h, respectively, for morning milking, and were 2.1, 1.8, and 1.7 h, respectively, for evening milking. On the other hand, for the gas measurement in the head box, cows in the experimental free‐stall barn were caught at the headlocks during feeding at 13:00 h and brought to the experimental barn equipped with head boxes. Therefore, gas measurements in the head box were performed immediately after eating. This time interval was much shorter than at the AMS measurement in both morning milking and evening milking. It is known that CH_4_ emission is high after eating, which is caused by activated fermentation in the rumen. Brask et al. (2015) observed a diurnal pattern of hourly CH_4_ emissions together with total SCFA concentration in the rumen, which peaked at 2 h after feeding, and also observed a reverse pattern for acetate molar proportion and pH in the rumen. Lassen et al. (2012) determined diurnal changes in the CH_4_/CO_2_ ratio with the curve including sinusoid function, showing a higher CH_4_/CO_2_ ratio in the daytime and a lower one in the nighttime. van Engelen et al. (2018), Pszczola et al. (2017), and Aguerre et al. (2011) also found similar diurnal patterns. The present results that the CH_4_/CO_2_ ratio decreased with time after eating (Table 4) agreed with their results.

**TABLE 4:** Results of linear mixed‐effects models on CH_4_/CO_2_ ratio, background‐corrected CH_4_ and CO_2_ concentration, and eructation rate in relation to the effects of time interval between gas measurement (milking) and the end of last eating before gas measurement, diet treatment, and the interaction effect of the time interval and diet treatment as variables

	CH_4_/CO_2_			CH_4_, %			CO_2_, %			Eructation, no./min		
	Coefficient	SE	*p* value	Coefficient	SE	*p* value	Coefficient	SE	*p* value	Coefficient	SE	*p* value
Intercept	0.102	0.0069	0.005	0.0354	0.00537	0.022	0.347	0.0548	0.024	1.24	0.067	0.003
Time after eating, /h	−0.0034	0.00067	<0.001	−0.0014	0.00068	0.047	−0.005	0.0075	0.525	−0.026	0.0108	0.017
Time × diet, /h												
LF	0.00015	0.001254	0.907	0.00012	0.001269	0.923	−0.008	0.0141	0.568	−0.015	0.0202	0.467
MF	0.00015	0.001102	0.892	0.00081	0.001121	0.469	0.015	0.0124	0.228	0.035	0.0179	0.050
HF	Reference			Reference			Reference			Reference		
Diet treatment												
LF	−0.026	0.0044	<0.001	−0.0081	0.00442	0.068	0.030	0.0491	0.544	−0.051	0.0705	0.467
MF	−0.016	>0.0012	<0.001	−0.0089	0.00430	0.041	−0.058	0.0478	0.225	−0.139	0.0687	0.044
HF	Reference			Reference			Reference			Reference		

*Note*. Squares and cows within squares were included as random effects.

Abbreviations: HF, high‐fiber; LF, low‐fiber; MF, medium‐fiber; SE, standard error.

The CH_4_/CO_2_ ratios in the AMS, assumed to be immediately after eating, calculated from the model in Table 4 applying 0 h as the time interval after eating by individual cows, were closer to those in the head box compared with the original CH_4_/CO_2_ ratio in the AMS without adjusting the time interval (Figure 3a,b). However, the slope of the linear regression equation passing through the origin between the CH_4_/CO_2_ ratio in the head box (X) and that in the AMS adjusted at 0 h after eating (Y) was 0.92, showing that the values in the AMS immediately after eating were still 8% lower than those in the head box (Figure 3b). This inconsistency in the CH_4_/CO_2_ ratio between the AMS and head box indicates the existence of other factors affecting the lower CH_4_/CO_2_ ratio in the AMS. One of the factors might be the diffusion effect in the AMS; clearer and larger oscillations in CH_4_ concentration, which mainly originated from eructation, were found during gas measurements in the AMS compared with the measurements in the head box, because of external diffusion in the AMS.

**FIGURE 4:**
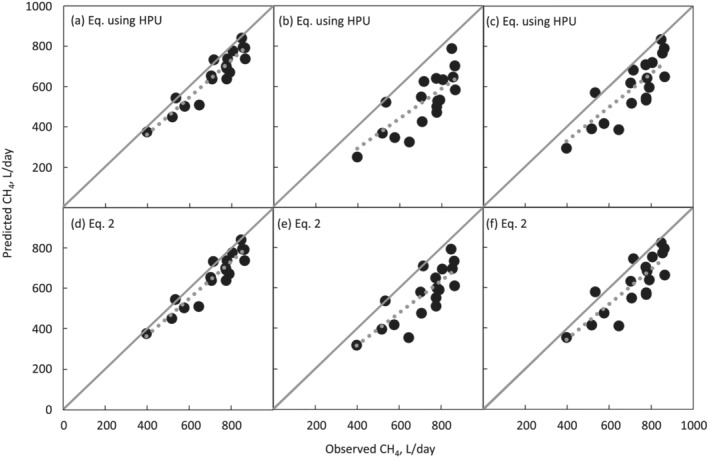
Relationships between observed (X) and predicted CH_4_ (Y) with the equation using HPU (a–c) and equation 2 (d–f) (*n* = 18). CH_4_/CO_2_ ratio in the head box was used for prediction in (a) and (d), and the ratio in the AMS was used in (b) and (e). CH_4_/CO_2_ ratio in the AMS of which time interval after eating was adjusted to 0 h, was used in (c) and (f). The equations were Y = 0.90X (a) (adjusted *R* ^2^ = 0.995, *p* < 0.01, RMSE = 47.9), Y = 0.74X (b) (adjusted *R* ^2^ = 0.975, *p* < 0.01, RMSE = 86.5), Y = 0.83X (c) (adjusted *R* ^2^ = 0.981, *p* < 0.01, RMSE = 84.7), Y = 0.92X (d) (adjusted *R* ^2^ = 0.995, *p* < 0.01, RMSE = 45.6), Y = 0.79X (e) (adjusted *R* ^2^ = 0.981, *p* < 0.01, RMSE = 81.1), and Y = 0.87X (f) (adjusted *R* ^2^ = 0.985, *p* < 0.01, RMSE = 78.0)

For the equation using the HPU and the selected Equation 2, the CH_4_ emissions that were predicted using the CH_4_/CO_2_ ratio in the head box matched well with the observed CH_4_ emissions in the head box (Figure 4a,d), indicating high reliability of the prediction equations. On the other hand, the CH_4_ emissions predicted using the CH_4_/CO_2_ ratio in the AMS were lower than the observed CH_4_ emissions, resulting from the lower CH_4_/CO_2_ ratio in the AMS than that in the head box (Figure 4b,e), as discussed above. On the other hand, the CH_4_ emissions predicted using the adjusted CH_4_/CO_2_ ratio at 0 h after eating were closer to the observed values than were the predicted CH_4_ emissions without adjustment (Figure 4c,f). In the present study, only the time interval between the last meal and gas measurement was focused, but feed intake during the last meal would also affect rumen fermentation, and consequently CH_4_ emissions. Further studies will be necessary to evaluate the effect of meal size on CH_4_ emissions.

### 3.3 Model development and evaluation for the prediction of MCF

**FIGURE 5:**
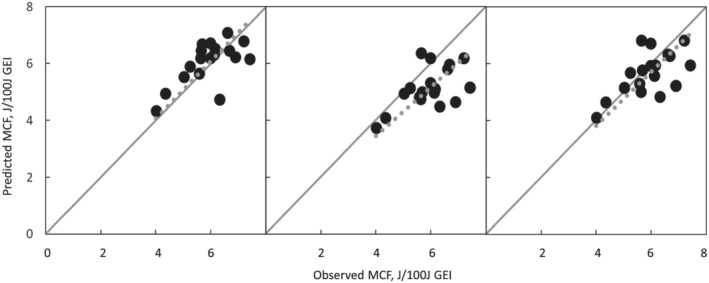
Relationships between observed MCF and predicted MCF with equation 10 (*n* = 18). The variable of CH_4_/CO_2_ ratio in each equation was obtained from measurements in the head box (a) or in the AMS (b). CH_4_/CO_2_ ratio in the AMS of which time interval after eating was adjusted to 0 h was used in (c). The equations were Y = 1.0X (a) (adjusted *R* ^2^ = 0.986, *p* < 0.01, RMSE = 0.72), Y = 0.86X (b) (adjusted *R* ^2^ = 0.981, *p* < 0.01, RMSE = 0.71), Y = 0.95X (c) (adjusted *R* ^2^ = 0.984, *p* < 0.01, RMSE = 0.72). MCF, CH_4_ conversion factor (CH_4_ energy in joules [J]/100 J of gross energy intake [GEI])

The addition of DMI or ECM to the MCF model consisting of the CH_4_/CO_2_ ratio as the sole variable (Equation 12 in Table 2) resulted in a marked improvement in model fitness (Equations 10 and 11). The coefficient of LW in the model consisting of the CH_4_/CO_2_ ratio, ECM, and LW was not significant (*p* = 0.407, adjusted *R* ^2^ = 0.603, RMSE = 0.573). The addition of LW to the model consisting of the CH_4_/CO_2_ ratio and DMI as a variable (Equation 11) resulted in a slight improvement in model fitness (Equation 9). Only a few improvements in model fitness with the addition of the ECM to Equation 9 were due to the high correlation between the DMI and ECM (Equation 8). This also resulted in the inclusion of a relatively higher VIF in Equation 8 (the highest VIF was 3.8 for DMI), whereas VIFs in other equations for MCF were below 1.4. Shibata et al. (1993) found that CH_4_ emissions per DMI decrease with increasing DMI, whereas CH_4_ emissions increase. This result shows that increasing the DMI leads to a decrease in the retention time of rumen digesta and a reduction in digestibility, and finally a reduction in CH_4_ emission efficiency. The coefficients for the independent variables of DMI were negative in the equations for MCF prediction (Table 2), indicating a negative effect of DMI on CH_4_ emission efficiency. Yan et al. (2010) and Ramin and Huhtanen (2013) also found negative coefficients for an independent variable of GEI per metaboli LW or DMI per LW in the prediction equation for MCF. From the perspective of model fitness, a lower degree of multicollinearity in the models, and convenience in determination on the commercial farm, Equation 10, which requires only ECM along with the CH_4_/CO_2_ ratio, seemed to be useful for predicting MCF. The predicted MCF using the CH_4_/CO_2_ ratio (Equation 10) in the head box matched well with the observed MCF, compared with the values predicted using the CH_4_/CO_2_ ratio in the AMS (Figure 5a,b). The underestimation of MCF was improved by using the adjusted CH_4_/CO_2_ ratio at 0 h after eating, as shown in Table 4 (Figure 5c).

### 3.4 Detection of dietary effect and variation of eructation

Aguerre et al. (2011) found that CH_4_ emissions from cows increased with the forage ratio or NDF content in the TMR. The prediction equations, including the positive coefficient for the independent variable of dietary NDF content, have been proposed for daily CH_4_ emission (Ramin & Huhtanen, 2013) and CH_4_ emissions per DMI (Moraes et al., 2014) in lactating cows, indicating the primary role of carbohydrate sources in rumen fermentation and CH_4_ production. The increase in the acetate‐to‐propionate ratio and milk‐fat content with increasing dietary NDF content supports the result of increasing observed CH_4_ emissions in the head box (Table 3). For Equations 1–4 and 10 and the equation using HPU, predicted CH_4_ emissions, or MCF using the CH_4_/CO_2_ ratio in the AMS corrected at 0 h after eating, were also affected by the dietary NDF content (Table 5). These results show that an increase in CH_4_ emission with increasing NDF content could be detected in the present study using six cows and diets with NDF content ranging from 32% to 47%. Although there is a potential for underestimation of CH_4_ emission prediction as mentioned above, the relative order is similar to that observed. Therefore, Equations 1–4 and 10 and the equation using HPU seem possible for evaluating the effect of diet on CH_4_ emissions.

**TABLE 5:** Observed and predicted daily CH_4_ emissions and CH_4_ conversion factors (MCF) of the cows fed high‐fiber (HF), medium‐fiber (MF), or low‐fiber (LF) diets

	Variables	LF	MF	HF	SEM	*p* value
CH_4_ emission, L/day						
Observed^†^		618^b^	768^a^	765^ab^	50.1	0.033
Equation using HPU^‡^	CH_4_/CO_2_ ^§^, MLW, ECM, DIP	522^b^	592^ab^	669^a^	65.6	0.007
Equation 1	CH_4_/CO_2_, DMI, LW, ECM	611^B^	671^AB^	712^A^	51.7	0.067
Equation 2	CH_4_/CO_2_, LW, ECM	563^b^	618^ab^	678^a^	61.8	0.017
Equation 3	CH_4_/CO_2_, LW, DMI	621^B^	681^AB^	717^A^	47.1	0.088
Equation 4	CH_4_/CO_2_, ECM	524^b^	585^ab^	639^a^	59.3	0.027
Equation 5	CH_4_/CO_2_, DMI	614	678	708	46.4	0.120
Equation 6	ECM	570	577	557	27.6	0.530
Equation 7	DMI	668	692	661	29.7	0.541
MCF, J/100 J GEI						
Observed^†^		5.15^b^	6.17^a^	6.42^a^	0.306	0.008
Equation 10	CH_4_/CO_2_, ECM	4.06^b^	5.56^b^	6.41^a^	0.255	<0.001

*Note*: Values with different superscript lowercase letters differ significantly (*p* < 0.05). Values with different superscript uppercase letters tend to differ (0.05 ≤ *p* < 0.10).

Abbreviations: DIP, days in pregnancy; ECM, energy‐corrected milk; LW, live weight; MCF, CH_4_ energy in joules (J) per 100 J of gross energy intake (GEI); MLW, metabolic live weight; SEM, standard error of the mean.

^†^ Observed in head box.

^‡^ Derived from the equation proposed by Madsen et al. (2010).

^§^ CH_4_/CO_2_ ratios in the AMS of which the time interval after eating was adjusted to 0 h using the model for the CH_4_/CO_2_ ratio in Table 4.

Variation of eructation rate in the present study (averaging 1.1 ± 0.13 [SD] and ranged from 0.9 to 1.4) was in the general range of 0.7 and 1.5 times per minute (summarized by Pickering et al., 2015). Rumen motility, which is closely related to eructation rate, is more active during eating than that during rumination or resting (Waghorn & Reid, 1983), and it is affected by the physical property of the feed (Nørgaard, 1989), though not by the variety of the feed (Waghorn & Reid, 1983) or feed intake (Ulyatt et al., 1984). The roughage value index, expressed as total chewing time per DMI, was similar among diets, but longer eating time per DMI, and higher forage ratio of HF diet compared with MF and LF diet may indicate a higher physical effect of HF diet (Table 3). This higher physical effect and higher NDF content might result in a higher eructation rate of cows fed the HF diet compared with cows fed the MF diet (Table 4). However, these effects were not observed from the comparison between cows fed HF and LF diet. For the cows fed the HF and LF diets, the model in Table 4 showed a decreasing eructation rate with time after eating (−0.03/h and −0.04/h, respectively), reflecting a decrease in rumen digesta and a consequent decrease in rumen motility. For the cows fed the MF diet, the model showed an increasing eructation rate with time after eating, but this elevation was very low (0.01/h). The effect of diet on the eructation rate was not clear, but the observed eructation rates were in the normal range, indicating that the present measurement seems to provide an appropriate eructation rate. Additionally, the detection of eructation rate could be used to estimate the physiological status of cows.

In conclusion, to predict CH_4_ emissions, the best fit and most useful models requiring no DMI measurement were the equations using HPU and Equation 2, which do not require reproduction information. On the other hand, careful consideration is needed for the CH_4_/CO_2_ ratio to eliminate the effect of the time interval after eating and derive the daily average. Intervals of feeding, measurements at various times of day, multiple measurements, or data correction using the present model based on time after eating or Fourier series approach (Løvendahl & Bjerring, 2006) would put the predicted value closer to the representative value. MCF seems to be predictable using the CH_4_/CO_2_ ratio and ECM as variables in the present equation without the measurement of GEI (Equation 10), but it was also affected by diurnal variations in the CH_4_/CO_2_ ratio. The combination of the sniffer method and proposed models should be available for the evaluation of the effect of diet on CH_4_ emissions or for the evaluation of CH_4_ emission inhibitors.

## CONFLICT OF INTEREST

All authors declare that they have no conflicts of interest.

## Supporting information


**Table S1** Information for individual studies
Table S2 Characteristics of the dataset used for model development
Diagram S1. Measurement system of methane to carbon dioxide ratio in the breath during milking.
Figure S1 Diurnal changes in average eating time for each hour of each treatment. Black, dotted and grey lines show eating time in cows fed LF, MF or HF diet, respectively. The diets were offered at 10:00 h, 13:00 h, and 17:00 h. Black and grey two‐way arrow show duration of gas measurement in the head box and milking time permitted, respectively.
