# Supplementary material for: Prediction of enteric methane emissions from lactating cows using methane to carbon dioxide ratio in the breath
Source: Anim Sci J. 2021 Sep 30;92(1):e13637. doi: 10.1111/asj.13637 (PMC9285552; doi:10.1111/asj.13637)
Supplement: Supplementary file 1 — Table S1 Information for individual studies Table S2 Characteristics of the dataset used for model development Diagram S1. Measurement system of methane to carbon dioxide ratio in the breath during milking. Figure S1 Diurnal changes in average eating time for each hour of each treatment. Black, dotted and grey lines show eating time in cows fed LF, MF or HF diet, respectively. The diets were offered at 10:00 h, 13:00 h, and 17:00 h. Black and grey two‐way arrow show duration of gas measurement in the head box and milking time permitted, respectively. [file ASJ-92-e13637-s001.pdf]

Estimation of enteric methane emission from lactating Holstein cows using the methane to carbon dioxide ratio in the breath

SUZUKI Tomoyuki, KAMIYA Yuko, OIKAWA Kohei, SHINKAI Takumi, NONAKA Itoko, OBITSU Taketo, TERADA Fuminori

Supporting information

Table S1 Information for individual studies

References	n	Apparatus	Temperature & relative humidity	Diets
Terada & Muraoka (1994)	32	Chamber	18 °C & 60% or 28 °C & 60%	Timothy hay, alfalfa hay cube, steam-rolled corn, soybean meal
Shioya et al. (1997)	15	Chamber	18 °C & 60%, 28 °C & 40%, or 28 °C & 80%	Italian ryegrass, alfalfa hay cube, barley or steam-rolled corn, soybean meal
Shioya et al. (1997)	14	Chamber	28 °C & 60% or 24-32 °C & 40-80%	Bahiagrass hay, steam-rolled corn, soybean meal, fish meal*, calcium soap of fatty acids*
Shioya et al. (1997)	8	Chamber	28 °C & 60%	Italian ryegrass, commercial concentrate
Terada et al. (Unpublished)	8	Head box	18 °C & 60% or 28 °C & 60%	Bahiagrass hay, steam-rolled corn, beet pulp
Nakai et al. (1999)	12	Chamber	18 °C & 60% or 28 °C & 60%	Bahiagrass hay, commercial concentrate
Terada et al. (Unpublished)	8	Chamber	18 °C & 60% or 28 °C & 60%	Italian ryegrass wafer, commercial concentrate
Nonaka et al. (2001)	16	Chamber	18 °C & 60% or 28 °C & 60%	Italian ryegrass wafer, steam-rolled corn, barley, soybean meal, fish meal*, beet pulp, alfalfa meal
Suzuki et al. (Unpublished)	8	Chamber	18 °C & 60%	Sudangrass silage, commercial concentrate

* The diet was used only on some groups within the study.

Reference of Table S1

Nakai, F., Hirashima, Y., Ueda, K., Pirnomoadi, A., Higuchi, K., Enishi, O., & Terada, F. (1999). Effect of dietary protein degradability on nitrogen and energy utilization of lactating cows. Animal Science Journal, 70, J390–J396. (In Japanese).

Nonaka, I., Itoh, F., & Purnomoadi, A. (2001). Energy metabolism in lactating cows treated with recombinant bovine somatotropin under high environmental temperature. In: Chwalibog, A., Jakobsen, K. (Eds.), Energy metabolism in animals: proceedings of the 15th Symposium on Energy Metabolism in animals. EAAP publication no. 103, Wageningen Academic Publishers, Wageningen, the Netherlands, pp. 397–401.

Shioya, S., Terada, F., & Iwama, Y. (1997). Physiological response of lactating cows under the hot environment. Proceedings of Japanese Society for Animal Nutrition and Metabolism, 41, 61–68. (In Japanese).

Terada, F., & Muraoka, M. (1994). Effect of heat stress on the efficiency of utilization of metabolizable energy for lactation. In: Aquilera, J. F. (Ed.) Energy metabolism of farm animals. EAAP publication No. 76, CSIC Publishing Service, Spain, pp. 323–326.

Table S2 Characteristics of the dataset used for model development

	Mean	SD	Minimum	Maximum
Parity	3.1	1.46	1.0	8.0
Day in milk	147	69.1	48	300
Live weight, kg	589	62.0	453	765
DMI, kg/day	16.7	3.68	6.1	26.0
ECM yield, kg/day	25.4	6.25	11.1	39.5
CH_4/CO_2 ratio, L/L	0.088	0.0119	0.044	0.108
CH_4 emission				
L/day	471	99.9	217	695
L/kg DMI	19.2	4.63	8.8	36.2
MCF, J/100 J GEI	6.12	0.909	3.40	7.98

n = 121; SD, standard deviation; DMI, dry matter intake; ECM, energy-corrected milk; MCF, methane conversion factor [CH4 energy in joules (J) per 100 J of gross energy intake (GEI)]

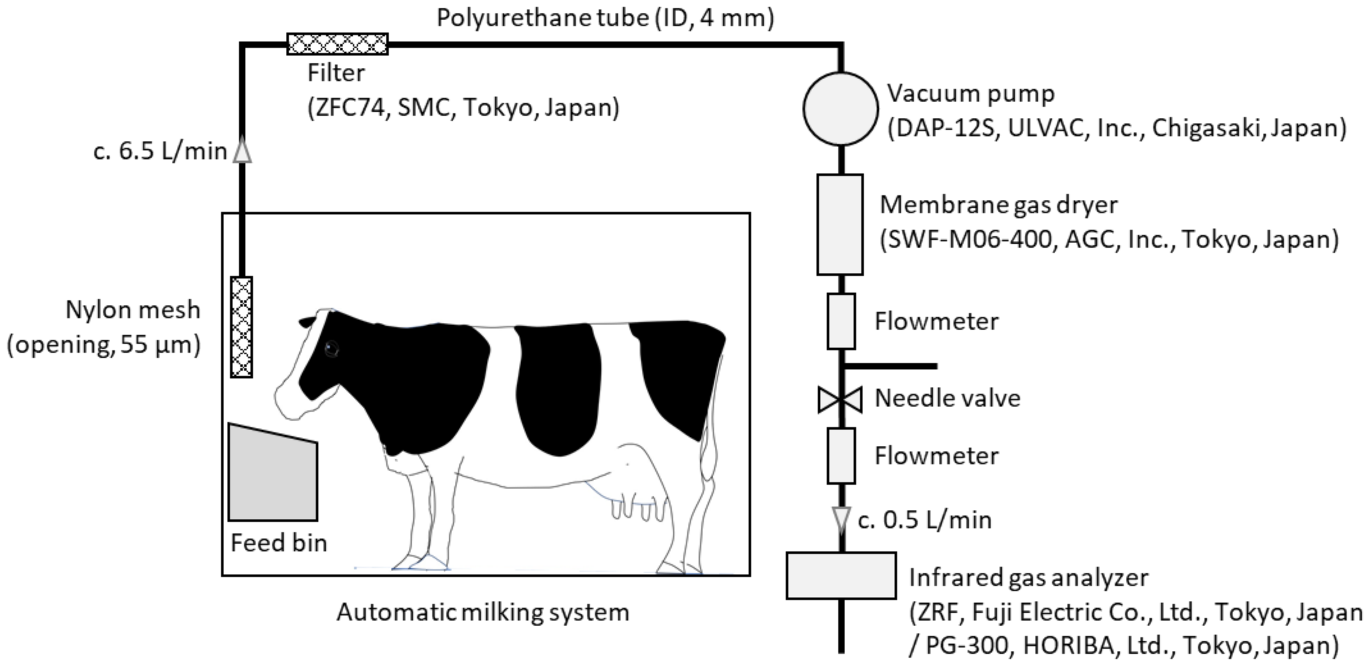

Diagram S1 Measurement system of methane to carbon dioxide ratio in the breath during milking.

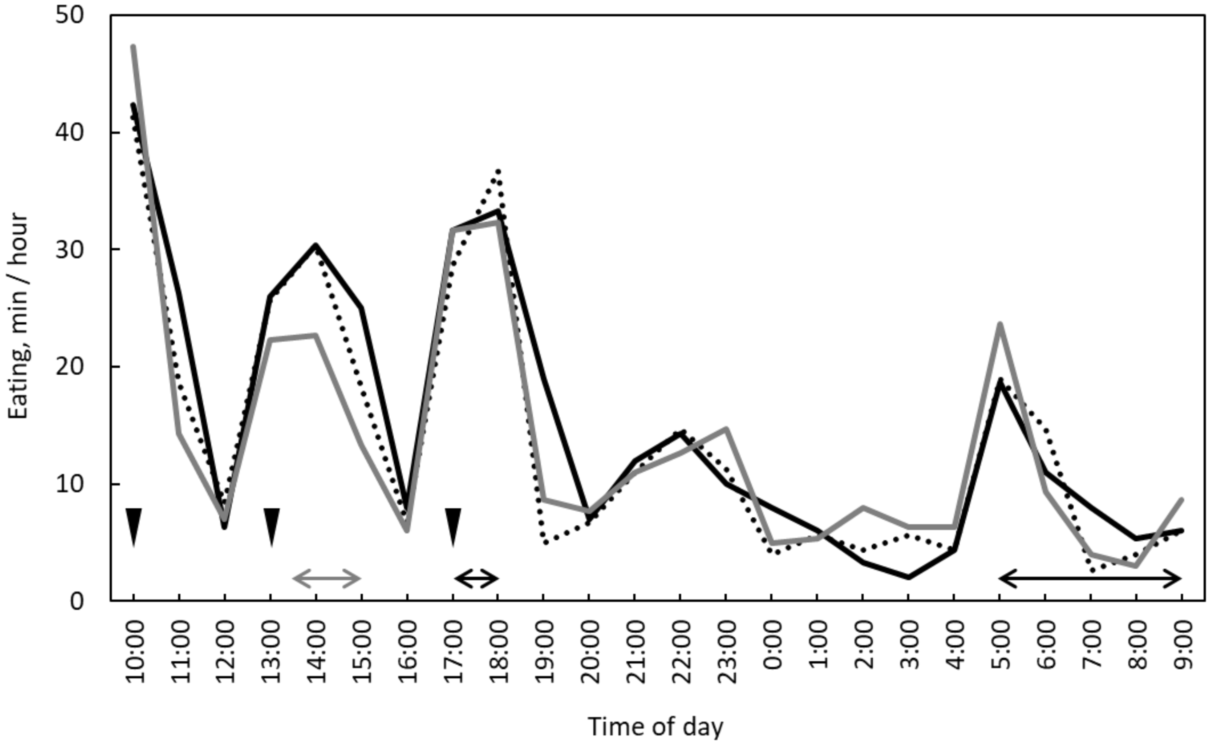

Figure S1 Diurnal changes in average eating time for each hour of each treatment. Black, dotted and grey lines show eating time in cows fed LF, MF or HF diet, respectively. The diets were offered at 10:00h, 13:00h, and 17:00h. Black and grey two-way arrow show duration of gas measurement in the head box and milking time permitted, respectively.
